# Single-cell transcriptomics reveal the heterogeneity and dynamic of cancer stem-like cells during breast tumor progression

**DOI:** 10.1038/s41419-021-04261-y

**Published:** 2021-10-21

**Authors:** Guojuan Jiang, Juchuanli Tu, Lei Zhou, Mengxue Dong, Jue Fan, Zhaoxia Chang, Lixing Zhang, Xiuwu Bian, Suling Liu

**Affiliations:** 1 Fudan University Shanghai Cancer Center & Institutes of Biomedical Sciences; Cancer Institutes; Key Laboratory of Breast Cancer in Shanghai; The Shanghai Key Laboratory of Medical Epigenetics; The International Co-laboratory of Medical Epigenetics and Metabolism, Ministry of Science and Technology; Shanghai Medical College; Fudan University, 200032 Shanghai, China; 2 Singleron Biotechnologies, Yaogu Avenue 11, 210043 Nanjing, Jiangsu China; 3 Institute of Pathology and Southwest Cancer Center, Southwest Hospital, Third Military Medical University (Army Medical University); Key Laboratory of Tumor Immunopathology, Ministry of Education of China, 400038 Chongqing, China

**Keywords:** Breast cancer, Cancer stem cells

## Abstract

Breast cancer stem-like cells (BCSCs) play vital roles in tumorigenesis and progression. However, the origin and dynamic changes of BCSCs are still to be elucidated. Using the breast cancer mouse model MMTV-PyMT, we constructed a single-cell atlas of 31,778 cells from four distinct stages of tumor progression (hyperplasia, adenoma/MIN, early carcinoma and late carcinoma), during which malignant transition occurs. We identified that the precise cell type of ER^low^ epithelial cell lineage gave rise to the tumors, and the differentiation of ER^high^ epithelial cell lineage was blocked. Furthermore, we discovered a specific signature with a continuum of gene expression profiles along the tumor progression and significantly correlated with clinical outcomes, and we also found a stem-like cell cluster existed among ER^low^ epithelial cells. Further clustering on this stem-like cluster showed several sub-clusters indicating heterogeneity of stem-like epithelial cells. Moreover, we distinguished normal and cancer stem-like cells in this stem-like epithelial cell cluster and profiled the molecular portraits from normal stem-like cell to cancer stem-like cells during the malignant transition. Finally, we found the diverse immune cell infiltration displayed immunosuppressive characteristics along tumor progression. We also found the specific expression pattern of cytokines and their corresponding cytokine receptors in BCSCs and immune cells, suggesting the possible cross-talk between BCSCs and the immune cells. These data provide a useful resource for illuminating BCSC heterogeneity and the immune cell remodeling during breast tumor progression, and shed new light on transcriptomic dynamics during the progression at the single-cell level.

## Introduction

Breast cancer is one of the most malignant cancers that seriously threat women’s health and cause casualties [1] and a malignancy with a multistep pathological processes starting with the premalignant atypical ductal hyperplasia (ADH), followed by ductal carcinoma in situ (DCIS) and subsequent malignant invasive ductal carcinoma (IDC) [2]. The full spectrum of distinct cell types and their molecular characteristics during the breast cancer tumorigenesis remain to be well studied, especially at the single-cell level. The mammary tumors developed in MMTV-PyMT breast cancer mice mainly go through four stereotypical stages, including hyperplasia at 4 to 6 weeks of age, adenoma/mammary intraepithelial neoplasia at 8–9 weeks of age, early malignant between 8–12 weeks of age and late-malignant at 13 weeks later, respectively [3]. This progress in mouse model mirrors the pathological procession of human breast cancer patients, and is comparable to human breast diseases classified as benign or in situ proliferative lesions to invasive carcinomas [3].

Breast cancer stem-like cells (BCSCs) are a rare subpopulation of tumor cells characterized with strong tumorigenic capacity. A serial of evidence supported BCSCs as the origin of breast cancer [4–6]. Recent investigations revealed that BCSCs are clinically, molecularly, and biologically heterogeneous [7–9]. However, accumulating evidence has shown that the heterogeneity of BCSCs based on the limited known markers is underestimated, suggesting the existence of more subsets of BCSCs [7]. The single-cell RNA-sequencing technology (scRNA-seq), which emerged in recent years, has played an increasingly important role in biological research [10–12]. Nowadays, scRNA-seq has been widely used in the research of tumor heterogeneity, immune microenvironment, neuroscience, embryonic development, cell differentiation, and others [13,14]. Furthermore, scRNA-seq has proved its power in revealing rare subpopulations [15–17].

Here, we provided the transcriptome analysis of 31,778 single cells including epithelial and immune cells from four different tumor progression stages of MMTV-PyMT breast cancer mouse model. We identified that the precise cell type of ER^low^ epithelial cell lineage gave rise to the tumors. We also characterized the stem-like cell cluster, and further clustering on this stem-like cell cluster showed several sub-clusters, indicating heterogeneity of stem cells. These results provided evidence that BCSCs are transcriptionally and functionally heterogeneous at the single-cell level.

## Results

### Single-cell transcriptome charted cell heterogeneity in MMTV-PyMT mouse mammary glands

**Fig. 1:**
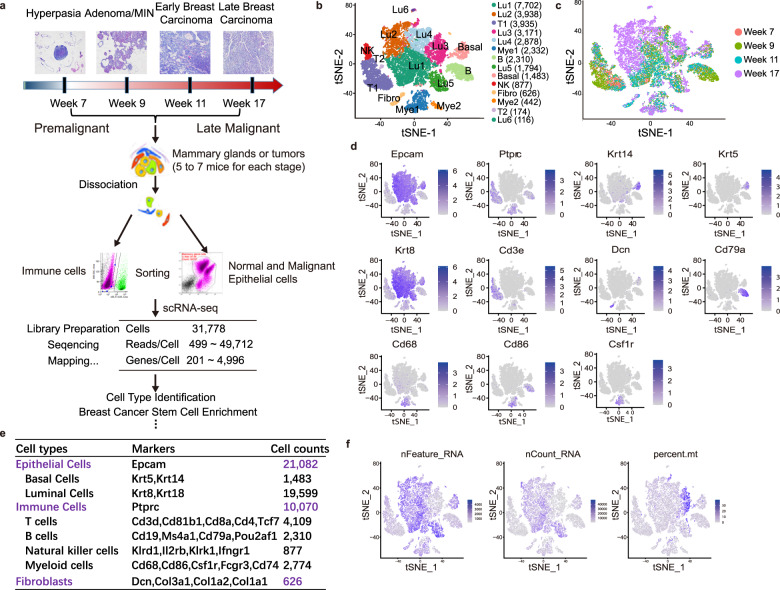
Single-cell analysis of mammary glands and tumors from MMTV-PyMT mice. **a** A schematic diagram highlighting the workflow including isolation and sequencing of single cells for this study. Single cells were prepared from the 4th pair of mammary glands of MMTV-PyMT mice at different tumor progression stages including hyperplasia, adenoma/mammary intraepithelial neoplasia (MIN, early carcinoma) and malignant tumors (late carcinoma). The transcriptome of single cells was sequenced using the 10x Chromium system. **b** The t-SNE plot of 31,778 single cells to visualize cell-type clusters based on the expression of known markers, Lu1, 2, 3, 4, 5, and 6: luminal epithelial cell cluster 1, 2, 3, 4, 5, and 6; Basal: basal epithelial cell cluster; Fibro: fibroblast cluster; T1 and 2: T cell cluster 1 and 2; B: B cell cluster; NK: natural killer cell cluster; Mye1 and 2: myeloid cell cluster 1 and 2. **c** The t-SNE plot of single-cell transcriptomes from the isolated cells at indicated tumor progression stages including week 7, week 9, week 11, and week 17. **d** The individual gene t-SNE plots showing the expression levels and distribution of representative markers of known cell types, which distinctly separates epithelial cells, immune cells and fibroblasts in the PyMT mouse mammary glands. **e** A table showed the markers used to annotate the known cell types, including epithelial cells, immune cells and fibroblasts in the PyMT mouse mammary glands. **f** The number of expressed genes (nFeature_RNA), the distribution of library size (nCount_RNA) and percentage of mitochondrial genes (percent.mt).

To characterize the single-cell transcriptome dynamics of MMTV-PyMT breast cancer mouse model during tumorigenesis, a total of 31,778 isolated single cells were obtained from mouse mammary glands or tumors, which spanned the cascade from hyperplasia to late breast carcinoma including premalignant, early malignant, and malignant stages. Then we obtained bulk transcriptomics from W07 (Week 7), W09 (Week 9), W11 (Week 11), and W17 (Week 17) mammary glands or tumors of MMTV-PyMT mouse model (Fig. 1a; Fig. S1a).

A total of 14 clearly separated cell clusters were finally identified (Fig. 1b, c). Based on the expression of known markers, we found that the cells are comprised mainly of clusters of epithelial cells, immune cells, and fibroblasts (Fig. 1d, e). As expected, the epithelial cells were largely basal epithelial cells and luminal epithelial cells, consistent with the cellular characteristics of mammary glands [18, 19]. In total, 19,599 luminal cells were clustered into six separate subsets (Lu1, Lu2, Lu3, Lu4, Lu5, and Lu6) (Fig. 1b). Lu2 and Lu4 were cancer cells, since they emerged almost uniquely in the late carcinoma of week 17, which were consistent with the previous reports that the PyMT mouse model has been characterized as most similar to the luminal B molecular subtype [20] (Fig. 1c). The immune cells comprised subsets of T cells including T1 and T2, B cells and myeloid cells including Mye1 and Mye2 (Fig. 1e). We also noticed that the number of expressed genes (nFeature_RNA), library size (nCount_RNA) and percentage of mitochondrial genes (percent.mt), the three commonly used quality controls in single-cell RNA sequencing, were also clearly distinguished between immune cells and epithelial cells (Fig. 1f). Since the sequencing quality of immune and epithelial cells was different, we determined to filter out low-quality cells by different criteria (Fig. S1b). After filtration, 12,039 epithelial cells and 8,954 immune cells were used for further analysis (Fig. S1c, d).

### ER^low^ luminal epithelial cells gave rise to the tumor cells during breast tumorigenesis

**Fig. 2:**
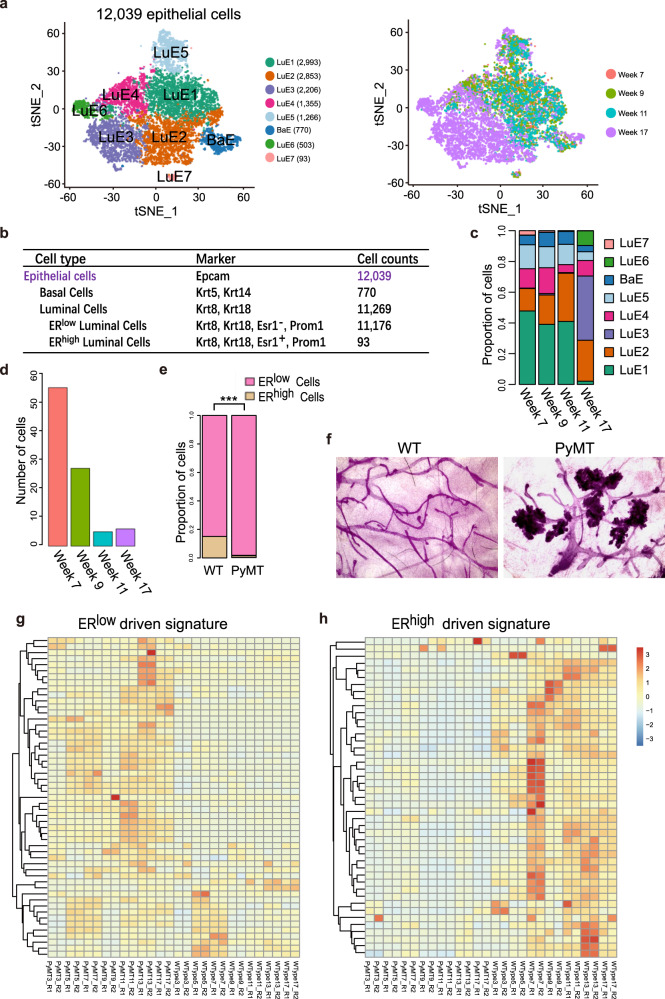
Tumor cells originated from ER^low^ luminal cells during tumorigenesis. **a** The t-SNE plot of 12,039 epithelial cells from the MMTV-PyMT mammary glands colored by cell types (left panel) and stages (right panel), week 7 (hyperplasia), 1,930 cells; week 9 (Adenoma/MIN), 2,886 cells; week 11 (early carcinoma), 2,038 cells; week 17 (late carcinoma), 5,185 cells. LuE1, 2, 3, 4, 5, 6, and 7: luminal epithelial cluster 1, 2, 3, 4, 5, 6, and 7; BaE: basal epithelial cluster. **b** Summary of cell counts and markers used for the identification of epithelial cell subsets from the mouse mammary glands and tumors. **c** The proportion of epithelial cell sub-clusters in different stages of tumor progression. **d** The number of ER^high^ epithelial cells across different stages of tumor progression dramatically decreased. **e** The ratio of ER^low^ and ER^high^ luminal epithelial cells in the mammary glands of MMTV-PyMT mice increased when compared to wild-type mice (WT), which suggests that the normal differentiation of ER^high^ cell lineage was blocked, resulting in the accumulation of ER^low^ cancer cells. **f** Whole mounts of mammary glands from wild-type (WT) and MMTV-PyMT mice indicated that cancer cells mainly exist in location of alveolar cells. **g** Gene expression profile of ER^high^ luminal signature was upregulated in epithelial cells from WT mice when comparing with epithelial cells from MMTV-PyMT mice. **h** ER^low^ luminal signature was highly expressed in MMTV-PyMT mouse epithelial cells relative to WT mice.

Breast cancer cells originate from epithelium in MMTV-PyMT mouse model, however, which subset of epithelial cells they originate from and the dynamic changes during tumorigenesis are still unknown. Thus, we re-clustered the filtrated 12,039 epithelial cells and obtained 8 clusters mainly comprised 7 luminal cell clusters termed LuE1, 2, 3, 4, 5, 6, and 7 (LuE, Luminal Epithelial cells) and one basal cell cluster termed BaE (BaE, Basal Epithelial cells) (Fig. 2a). 87.9% cells from 17 weeks were clustered together to form cluster LuE2, LuE3, LuE4, and LuE6. On the other hand, 69.7%, 62.3%, and 62.8% cells corresponding from 7, 9, and 11 weeks were clustered together to form cluster LuE1, LuE5, BaE, and LuE7, respectively (Fig. 2a–c). Cells were clearly separated between these two groups of clusters, indicating the unique transcriptional profile for the late carcinoma stage.

Previous studies have shown that luminal progenitor cells can develop into either ER^low^ or ER^high^ luminal progenitor cells, and subsequently differentiate into alveolar and ductal luminal cells [18]. We identified the cluster LuE7 as ER^high^ luminal cell cluster by the expression of Esr1 (Fig. 2b; Fig. S2a). We witnessed the percentage of ER^high^ luminal cells (LuE7) was decreased along the tumor progression (Fig. 2d) and there was a huge imbalanced percentage distribution of ER^low^ and ER^high^ luminal cells (Fig. 2e). 99.2% luminal cells were belonged to ER^low^ luminal cells. In order to distinguish the possibility of imbalanced distribution of ER^low^ and ER^high^ luminal cells due to the natural development, we compared the percentage of ER^low^ cells between MMTV-PyMT and wild-type FVB mice using the published dataset [21]. We identified ER^low^ and ER^high^ luminal cells (Fig. S2b, c) and counted the cell number in luminal epithelial cell clusters (Fig. S2d). ER^low^ cells were significantly enriched in our data while ER^high^ cells were depleted from MMTV-PyMT mouse when comparing to the FVB mouse data (Fig. 2e). Hence, the developmental process from luminal progenitor to ER^high^ luminal cells was blocked during tumorigenesis. Since the ER^low^ subpopulation are the major luminal cells, we proposed that the cancer cells in MMTV-PyMT mouse were mainly ER^low^ cells and derived from ER^low^ luminal progenitor cells. It is clear that the tumors are originated from the site of alveolar cells, which are reported as ER^low^ cells prepared for milk secretion once corresponding conditions and hormone stimulation exist (Fig. 2f). We compared the differentially expressed genes between the ER^high^ and ER^low^ luminal cells and defined the genes upregulated in ER^high^ luminal cell population as ER^high^ signature and the genes upregulated in ER^low^ luminal cell population as ER^low^ signature, respectively. Besides, we included several genes reported as marker genes for ER^high^ luminal cell by previous studies into the ER^high^ signature such as Esr1, Foxa1, Gata3, Pgr, ect [22] (Table S1). Then we investigated the expression profile of these two signatures in bulk RNA-seq from matched MMTV-PyMT and wild-type FVB mice. Most of ER^low^ signatures were highly expressed in MMTV-PyMT mammary tissues, while most of ER^high^ signatures were highly expressed in FVB counterparts (Fig. 2g, h). It was consistent with the imbalanced percentage distribution of ER^low^ and ER^high^ luminal cells.

Taken together, our data suggested that the specific cell-type ER^low^ luminal cells gave rise to tumors, and the differentiation of the ER^high^ cell lineage was blocked. The accumulation of proliferative ER^low^ was the fundamental origin of the cancer cells in MMTV-PyMT mouse.

### A continuum of gene expression profiles revealed the malignant transition of ER^low^ luminal cells in the mammary glands

**Fig. 3:**
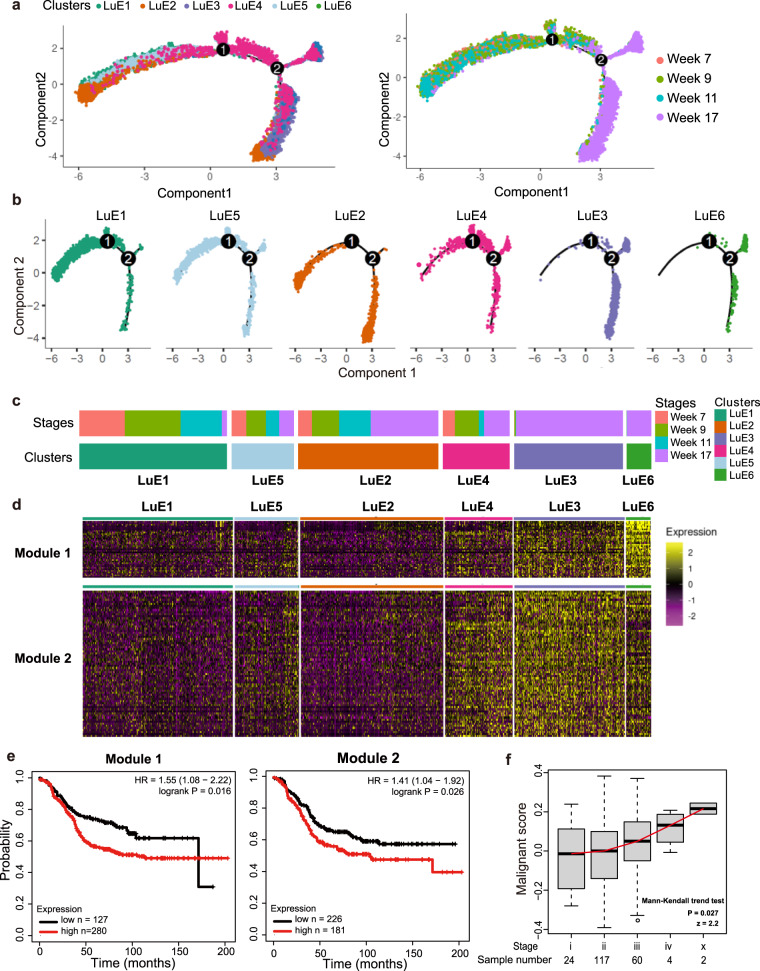
A continuous change of specific gene signature in ER^low^ luminal cells was correlated with the tumor progression and clinical outcome. **a** Pseudo-time trajectory analysis of the six sub-clusters of luminal epithelial cells annotated by clusters (left) and tumor progression stages (right). **b** Pseudo-time trajectory of cells in each luminal epithelial cell cluster. **c** The heatmap showed the sample composition of each cluster, indicating that the composition of week 17 was gradually increased along cluster LuE1, LuE5, LuE2, LuE4, LuE3, and LuE6. **d** Heatmap of expression profile of signature genes in indicated modules identified by WGCNA (Weighted Gene Co-expression Network Analysis). A summary list of genes associated with corresponding WGCNA module were shown in supplementary Table 3. **e** The Kaplan–Meier relapse free survival curves of patients were grouped by the gene signatures in WGCNA modules. The left corresponded to the module 1 and the right corresponded to the module 2. **f** Boxplot depicted the distribution of malignant score derived from luminal B patient samples in TCGA. Mann-Kendall trend test was performed using the median value of each stage by “trend” package in R.

Since we determined that the cancer cells originated from ER^low^ luminal cells, we then focused on the dynamic changes of these epithelial cells along the transitions from hyperplasia to late carcinoma. To this end, we constructed the single-cell trajectories to trace the ER^low^ luminal epithelial hierarchy during tumor progression (Fig. 3a). The genes used to construct single-cell trajectories was listed in Table S2. The location of each cell cluster showed a unique pattern on single-cell trajectories. The majority of cells from cluster LuE3 and LuE6 were located on one side of single-cell trajectories, while the cells from cluster LuE1 and LuE5 were mainly located on the other side. Moreover, cells from cluster LuE2 and LuE4 showed an intermediate state (Fig. 3b), suggesting the trajectories of the tumor progression along cluster LuE1, LuE5, LuE2, LuE4, LuE3, and LuE6. Indeed, the cluster of the trajectories was positively correlated with the percentage of cells from week 17, which are mainly cancer cells (Fig. 3c). To gain a more detailed view of gene expression pattern changes over the critical tumor progression and malignant transition, we performed weighted correlation network analysis among the different clusters to explore the specific gene expression pattern and we identified panels of specific signatures on the chronologic tumor progression, which matched the pattern derived from t-SNE plot and single-cell trajectories [23]. The expression of the genes in the modules was gradually increased from left to right along the clusters, composing elevated proportion of cells from week 17 (Fig. 3d). The genes in these two modules were listed in Table S3. Next, we found the significant correlation between genes from these two modules and the clinical outcome of Luminal B breast cancer patients in survival analysis, and patients with these highly expressed genes showed worse prognosis (Fig. 3e). Among these genes, we identified several related to tumor progression as reported in previous studies were also consistent with this pattern (Fig. S3a). Since these genes in both signatures were upregulated along the tumor progression with the similar pattern, we merged these two gene signatures and termed the combined signature as the malignant signature. We also explored the expression profile of malignant signature among different tumor grades in luminal B patient samples from The Cancer Genome Atlas (TCGA) [24]. The results showed that the malignant score was significantly increased in the higher tumor grades (*P* = 0.027) (Fig. 3f).

### Identification of BCSCs in ER^low^ luminal cells

There is increasing evidence to suggest that diverse solid tumors are hierarchically organized and may be sustained by distinct subpopulations of cancer stem-like cells (CSCs) [25, 26]. Here, we first characterized the heterogeneity of BCSCs at the single-cell level and then characterized the feature of these cells.

**Fig. 4:**
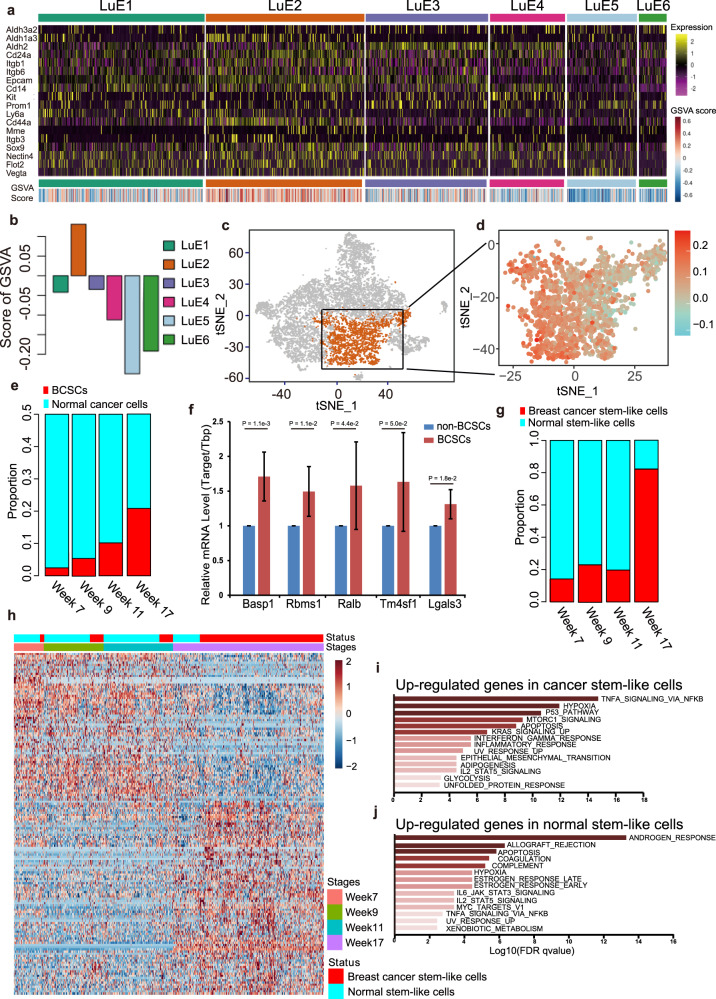
Identification and characterization of cancer stem-like cell cluster in ER^low^ luminal cells. **a** Heatmap of stem cell marker gene expression profile in each cell cluster (up panel) and GSVA score for each cell in each cell cluster (bottom panel). **b** A bar-plot showed the average GSVA score for each cell cluster. **c** The t-SNE plot showed the distribution of cancer stem-like cells enriched cluster LuE2 (orange, *n* = 2,206 cells) within the atlas. **d** The enlarged t-SNE plot highlighted the cells in cluster LuE2 colored by cancer signature score. **e** The proportion of BCSCs among cancer cells was defined by the median value of cancer signature score. Cancer cells were defined as the cells with cancer signature score higher than the median value. BCSCs were the cancer cells in LuE2. **f** Upregulated genes identified from our analysis were confirmed in CD24^+^CD29^+^ BCSCs from MMTV-PyMT mice by qPCR, compared to non-CD24^+^CD29^+^ BCSCs. The P was calculated by t-test. **g** The proportion of cancer stem-like and normal stem-like cells defined by the median value of cancer signature score. Cancer stem-like cells were defined as the cells with cancer signature score higher than the median value. Normal stem-like cells were defined as the cells with cancer signature score lower than the median value. **h** Overlap of the genes between cluster LuE2 marker genes and the differential expressed genes among normal cells and cancer cells in cluster LuE2 were shown by the unsupervised clustering heatmap. **i** Function enrichment analysis for the genes upregulated in cancer stem-like cells in Fig. 4h. **j** Function enrichment analysis for the genes upregulated in normal stem-like cells in Fig. 4h.

We searched the BCSC-related genes published previously, such as Cd24a, Itgb1, Itga6, Procr, etc in the literature and incorporated these genes into the BCSC signature (Fig. S3b). We scored each ER^low^ epithelial cell based on the BCSC signature by Gene Set Variation Analysis (GSVA) algorithm [27]. The expression profile of BCSC signature clearly showed a higher expression level on cluster LuE2 (Fig. 4a). Indeed, the score from GSVA algorithm was consistent with the expression profile (Fig. 4b). Since the cluster LuE2 was a mixture of cells from all four tumor progression stages, it is reasonable to assume that cluster LuE2 might include both normal and cancer cells. Next, we investigated the stemness score at different tumor progression stages and found no significant difference among these stages (Fig. S3c, d), suggesting the similarity of stemness in cluster LuE2 among tumor progression stages and the gene expression shared by both normal breast stem-like cells and BCSCs.

In order to distinguish between the normal and cancer cells, we extracted the up-/down-regulated genes comparing between bulk RNA-seq of PyMT and wild-type FVB mouse at week 17 as cancer signature genes to identify normal and cancer cells and the cancer signature genes can be found in Table S4. The results clearly showed a spatial pattern of normal and cancer cells on the t-SNE plot (Fig. 4c, d). The percentage of BCSCs among cancer cells was increased along the tumor progression which is consistent with previous studies (Fig. 4e) [28]. In order to confirm our analysis of the BCSC cluster, we identified the upregulated genes in the BCSC cluster compared to the non-BCSC cluster and picked four of these genes for the verification by qPCR. The genes upregulated in the BCSC cluster can be viewed in Table S5. The results showed that these four genes were significantly upregulated in CD24^+^CD29^+^ tumor cells from MMTV-PyMT mice, which was compared with non-CD24^+^CD29^+^ tumor cells (Fig. 4f). The upregulated genes identified from our analysis were also upregulated in traditionally defined BCSCs, indicating the similarity of these two groups of BCSCs.

Our studies showed that the BCSCs among all stem-like cells were also enriched in the last stage during tumor progression (Fig. 4g). We identified genes showing specific expression pattern between normal and cancer stem-like cells, providing as novel potential biomarkers to mark cancer stem-like cells (Fig. 4h and Table S6). The functional enrichment analysis revealed that these differentially expressed genes were enriched in the various important pathways including TP53, EMT and apoptosis pathways (Fig. 4i, j). In summary, our data revealed both the similarity and dissimilarity of gene expression between normal and cancer stem-like cells.

### BCSCs were heterogeneous with different biological function and transcription regulation

**Fig. 5:**
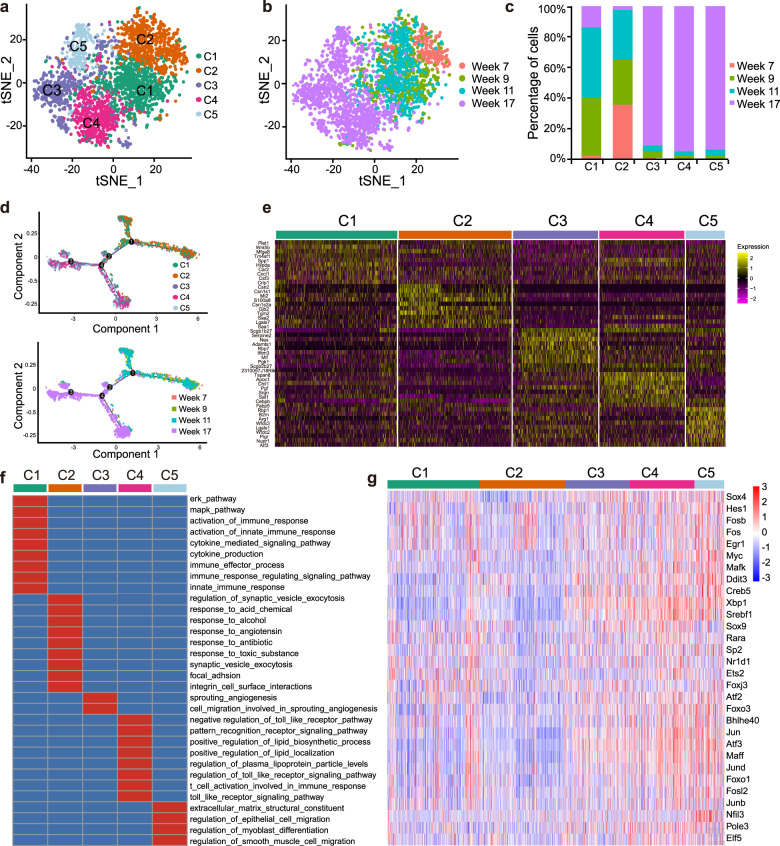
The heterogeneity of stem-like cells. **a**, **b** t-SNE plot demonstrated the separation of sub-clusters C1, C2, C3, C4, and C5 in LuE2 cluster colored by sub-clusters (**a**) and by tumor progression stages (**b**). **c** A bar-plot depicted the composition of cells from different tumor progression stages in each sub-cluster. **d** Pseudo-time analysis showed the single-cell trajectories for LuE2 cluster colored by sub-clusters (top panel) and tumor progression stages (bottom panel). **e** A heatmap showed the expression profile of marker genes from each sub-cluster. **f** A heatmap showed the function enrichment for each sub-cluster. Significant enrichment was colored by red. **g** A heatmap of regulon scores from SCENIC (Single Cell rEgulatory Network Inference and Clustering) analysis. Rows, Individual regulons. Columns, cells organized according to re-clustering of cluster LuE2.

It’s well known that tumors are highly heterogeneous [5, 29, 30]. Recent studies reported that the plasticity and heterogeneity are features of CSCs [31–36], but the specific heterogeneous BCSC populations and related markers are still unclear. In order to reveal the heterogeneity of BCSCs, we re-clustered the stem-like cell cluster LuE2 in a more fine-scale into five sub-clusters C1–C5 (Fig. 5a, b). We also checked the composition of cells from each tumor progression stage in the t-SNE plot and found that sub-clusters C1 and C2 existed across week 7, 9, and 11, and were mainly consisted of normal stem-like cells. However, sub-cluster C3, C4 and C5 existed almost mainly at week 17 and were mainly consisted of BCSCs (Fig. 5b, c). There was a clear separation between normal stem-like cells and BCSCs. To illuminate the possible evolution from the normal stem-like cell clusters to BCSC clusters, we also checked the single-cell trajectories and the inferred cell trajectory suggested a branched structure with sub-clusters from week 7, 9, and 11 positioned at the opposite end of the sub-clusters from week 17 (Fig. 5d). Next, we explored marker genes that were uniquely expressed in each sub-cluster (Fig. 5e), and performed functional enrichment analysis by Enrichr [37, 38] and the results showed specific functions were enriched in each sub-cluster, suggesting the functional heterogeneity in BCSCs. For example, genes uniquely expressed in C3 sub-cluster were enriched in cell migration and angiogenesis pathways (Fig. 5f). Then we investigated the transcriptional regulons based on cis-regulatory motif over the different clusters utilizing SCENIC analysis [39] and identified serials of transcription factors (TFs) correlated with each sub-cluster (Fig. 5g). Among these TFs, we found various TFs functioned in cancer/normal stem-like cells such as Sox4, Foxo3 and Myc, indicating their potential regulation on BCSC heterogeneity [40–42].

### The heterogeneity of BCSCs was confirmed in breast cancer patients

**Fig. 6:**
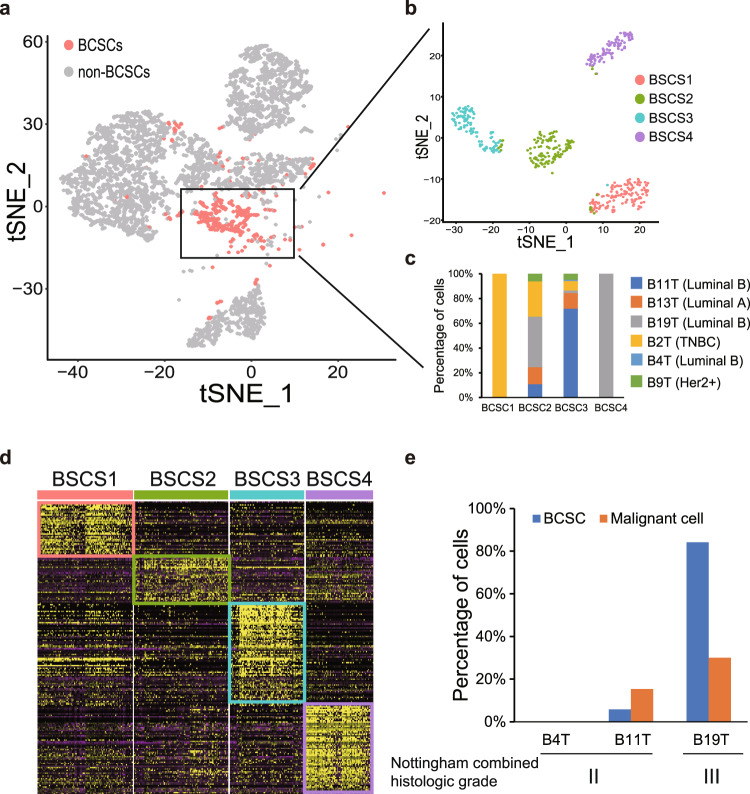
The heterogeneity of stem-like cells in breast cancer patient tumors. **a** The t-SNE plot highlighted the distribution of cancer stem cells from breast cancer patients. Cancer stem cells were highlighted in color and normal cancer cells were in gray. **b** Re-clustering of cancer stem cells by Seurat. **c** Histogram showed the composition of cancer stem cells from each patient. **d** Heatmap demonstrated the genes specifically expressed in each BCSC cluster. **e** Histogram showed the percentage of BCSCs and malignant cells in each patient.

In order to verify the heterogeneity of BCSCs observed in mouse scRNA-seq data, we collected tumor samples from six breast cancer patients which were classified as luminal A, luminal B, TNBC and Her2^+^ subtypes and performed the scRNA-seq analysis (Table S7). Totally, we obtained 8,990 cells after quality control and performed clustering to define the population structure. 13 clusters were identified including epithelium, immune, stroma, endothelial and other cell clusters (Fig. S4a) [43]. We extracted epithelium cells and scored each epithelial cell cluster based on the BCSC gene signature. The EP8, EP11 and EP12 were identified as BCSC clusters (Fig. S4b, c). The cells from these three clusters were extracted and re-clustered (Fig. 6a), and the t-SNE plot showed the cells were clearly separated into four groups which we named as BCSC1, BCSC2, BCSC3, and BCSC4 (Fig. 6b). Almost all cells in BCSC1 and BCSC4 were from B2T (TNBC) and B19T (Luminal B), respectively. On the other hand, the BCSC2 and BCSC3 were heterogenous clusters comprising the cells from all six patients (Fig. 6c). Furthermore, the cells in each BCSC cluster express a set of specific genes, reflecting the diversity of BCSCs (Fig. 6d).

Finally, we extracted the epithelium cells from three luminal B patients and evaluated the BCSC and malignant level by scoring based on the BCSC and malignant gene signature (Fig. S4d, e). We selected the clusters with high BCSC and malignant scores and summarized the percentage of cells within the BCSC and malignant clusters. The percentage of BCSC and malignant cells were very low/moderate in B4T/B11T patients. In contrast, both the percentage of BCSCs and malignant cells was high in B19T patient (Fig. 6e). Patient with higher percentage of BCSCs and malignant cells got higher score for histological grade which indicated worse clinical outcome [44].

### BCSCs cross-talked with the immune cells through cytokine signals to promote tumor progression

**Fig. 7:**
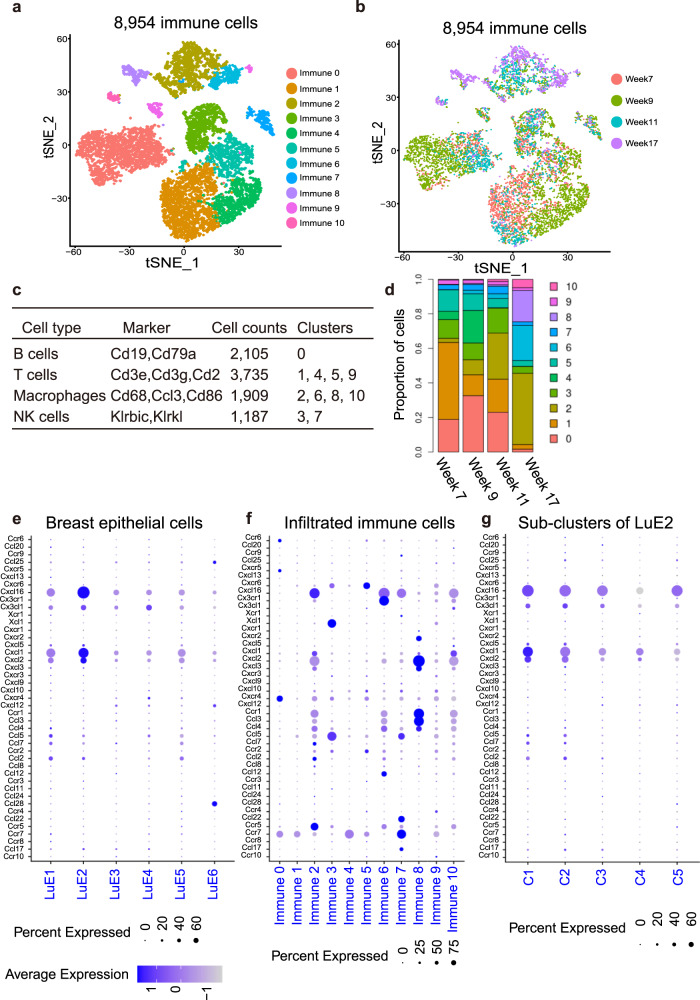
The cross-talk between BCSCs and immune cells during tumor progression. **a**, **b** The t-SNE plot showed the distribution of the immune cells from different clusters supervised by Seurat (**a**) and different tumor progression stages (**b**) in the atlas. **c** A table showed the markers used to annotate immune cells and cell accounts of the corresponding cell type. **d** Dynamic changes of the proportion of the immune cell types spanning from premalignant to late carcinoma. **e**–**g** Dot plots showed the expression of paired cytokine and cytokine receptors in ER^low^ clusters, immune clusters and sub-clusters of LuE2, which indicated that cytokine Cxcl1 and Cxcl16 were highly expressed in sub-clusters of LuE2 and the corresponding receptors Cxcr2 and Cxcr6 were highly expressed in macrophages and T cells.

BCSCs are critically regulated by the surrounding microenvironment, especially immune cells [45]. In order to explore the possible cross-talk between the BCSCs and the immune cells, we firstly identified and annotated various immune cell types and the dynamic changes along breast tumor progression in MMTV-PyMT mice. By re-clustering the immune cells, we obtained 11 clusters (Fig. 7a, b), which came from four categories including T cells, B cells, macrophages and natural killer cells (NK cells) (Fig. 7c; Fig S5a–d). We observed that the proportion of the immune cells including B cells, T cells, NK cells, decreased along the tumor progression (Fig. 7d). On the other hand, in clusters 6 and 8, macrophages were almost specifically shown in week 17. To systematically study the interactions between cancer stem-like cells and immune cells, we used the known repository of cytokine and cytokine-receptor interacting pairs that account for the interactions and considered the expression levels of ligands and receptors within each cell type. We found that cancer stem-like cell cluster LuE2 highly expressed Cxcl1, while the immune cells from cluster 8 defined as macrophages specifically highly expressed the corresponding receptor, Cxcr2 (Fig. 7e–f). Furthermore, LuE2 highly expressed Cxcl16 and T cell cluster 5 specifically highly expressed its receptor, Cxcr6 (Fig. 7e, f). Notably, when investigating the expression of the cytokine Cxcl16 and Cxcl1 in LuE2, we found that Cxcl16 showed relatively high expression in C1 (Fig. 7g). The vast majority of cells in C1 were from weeks 7, 9 and 11, indicating a strong immune response in the early stage of tumor development. This result was consistent with significant enrichment of immune functions such as activation of immune response, cytokine-mediated signaling pathway and cytokine production pathways in sub-cluster C1 of LuE2 shown in Fig. 5f. These studies indicated that BCSCs secreted different levels of cytokines among five sub-clusters and functioned through corresponding receptors on immune cells to promote tumor progression.

## Discussion

Taking advantage of scRNA-seq, we depicted the dynamic changes of transcriptional profile for epithelial and immune cells isolated from the mammary glands or tumors of MMTV-PyMT mouse model. To our knowledge, this is the first study to define a single-cell atlas of epithelial and immune cells during tumor progression in MMTV-PyMT mouse model.

The traditional method to isolate BCSCs is sorting by FACS based on cell-surface markers, but these BCSCs are usually a mixture of BCSC populations [6, 36]. Taking advantage of scRNA-seq combined with GSVA scoring, we identified the subpopulations of BCSCs and revealed the heterogeneity of BCSCs along the tumor progression. Re-clustering of cells from stem-like cell cluster LuE2 also confirmed the heterogeneity of stem-like cells.

Functional analysis showed that different stem-like cell sub-clusters enriched some unique functions such as the functions related to immune response for these sub-clusters, which may reflect the functional heterogeneity of cancer stem-like cells along tumor progression. These functions were uniquely enriched in the sub-cluster with late developmental stage reflected unique requirement for cancer stem-like cells. It will be helpful to gain insight into the functional requirement for cancer stem-like cells.

Evidence showed that BCSCs contribute to educate and reconstitute the immune microenvironment [46]. In our study, we found that the cancer stem-like cell cluster LuE2 secreted the specific cytokine Cxcl16 and Cxcl1. The cytokines can function through affecting and cross-talking with the corresponding immune cells. On the other hand, increasing evidence have demonstrated the role of immune microenvironment in the generation and maintenance of BCSCs [47]. Here, we revealed the dynamic changes of BCSC populations, where the proportion of immune cell types T, B, and NK cells decreased and the proportion of macrophages increased, which provided benefits for survival of the BCSCs.

Our findings provide unparalleled insight into the cellular heterogeneity of breast cancer with different types of premalignant lesions and malignant lesions, which may be helpful for identifying markers for cancer prevention and facilitate our understanding of breast cancer pathogenesis. Furthermore, our findings on the cross-talk of BCSCs and immune cells provide thoughts for the combination of immunotherapy and cancer stem cell targeted therapy for precise medicine.

## Methods and materials

### Breast cancer patient tumor tissues

All the breast cancer patient tumor tissues were obtained from Shanghai Cancer Hospital affiliated with Fudan University. An informed consent was obtained from all the involved patients, and the study was approved by the institution’s ethics committee (Fudan University Shanghai Cancer Center Institutional Review Board, 050432-4-1212B) (Shanghai, China).

### Mice and tissue collections

MMTV-PyMT and wild-type FVB mice were housed in standard animal cages under specific pathogen-free conditions in the Department of Laboratory Animal Science of Fudan University. Animal experiments were approved according to the experimental animal guidelines of the Care and Use of Laboratory Animals of Fudan University and approved by the Fudan University Shanghai Cancer Center Institutional Review Board (JS-082).

Mammary glands or tumors from 7-week old, 9-week old, 11-week old and 17-week old MMTV-PyMT FVB mice were excised, dissected and minced into small pieces and then resuspended with collagenase-hyaluronidase digestion reagent (Catalog #07912, STEMCELL Technologies, USA). Mammary glands or tumors for each stage were from 5 to 7 mice (seven mice for the first and second stages, and five mice for the third and fourth stages). The number of mice used in each experimental group was determined by power analysis and on the basis of prior experience with animal models. No mouse was excluded from the analyses and No randomization of mice was needed in this study. This study included a lot of complicated experimental design, the researchers were limited, and the feasibility of blinding was poor, thus blinding was not efficiently applied.

Tissue pieces were digested for approximately 1 hr at 37 °C and shaken once every 15 min. Cell aggregates were removed by filtering cell suspension with 40 μm filter. Cell suspensions were centrifuged at 1200 rpm for 5 m and resuspended for subsequent experiments.

### Fluorescent-activated cell sorting (FACS)

Dissociated cells from the mammary glands or tumors of MMTV-PyMT mice were suspended in FACS buffer containing anti-mouse cell lineage antibody cocktails: CD45 (1:50, 555483, BD), CD31 (1:50, 555446, BD), CD140b (1:50, 558821, BD), anti-CD24 (1:50, 138506, BioLegend, USA) and anti-CD29 (1:80, 102226, BioLegend). A MoFlo Astrios instrument (Beckman Coulter, Brea, USA) was used for sorting. Data acquisition and analysis were performed using Summit software.

Dissociated single cells were separately sorted by fluorescence-activated cell sorting (FACS) based on specific cell-surface markers. We used antibodies against endothelial marker CD31, b1-integrin CD29, heat-stable antigen CD24, hematopoietic marker CD140b and CD45 antigens to gate on the CD31^−^CD140b^−^CD45^−^ (Lineage^−^, Lin^−^) epithelial cell population including both normal and tumor epithelial cells and the CD31^−^CD140b^−^CD45^+^ immune cell population. Then, we defined four distinct Lin- epithelial cell subpopulations based on the expression of CD29 and CD24. The rare CD29^-^CD24^-^ population was excluded from the Lin- subpopulation since it was reported as the possible stromal population [19].

### Library preparation and sequencing for mouse sample

Single-cell sequencing was constructed using the 10x Genomics Chromium platform for droplet-enabled scRNA-seq according to the manufacturer’s instructions. Library generation was performed following the Chromium Single Cell 3′ Reagents Kits version 2 user guide in order to capture 5000 cells to 10000 cells/chip position (CG00052 Rev B). All the remaining procedures including the library construction were performed according to the standard manufacturer’s protocol. Each library was sequenced on the Illumina HiSeq 4000 platform to achieve an average of 48,488 reads per cell.

The raw data was processed by the Cell Ranger Single-Cell Software Suite (release 2.0), including using “cellranger mkfastq” to demultiplexes raw base call files into fastq-format files and then using “cellranger count” to perform reads alignment, filtering, barcode counting, and UMI counting. The reads were aligned to the mm10 reference genome using a pre-built annotation package downloaded from the 10X Genomics website. The output from different lanes was eventually aggregated using “cellranger aggr” with default parameters.

### Library preparation and sequencing for human sample

The fresh tumor tissue was stored in the GEXSCOPE^TM^ Tissue Preservation Solution (Singleron) and transported to the Singleron lab on ice as soon as possible. The specimens were washed with Hanks Balanced Salt Solution (HBSS) for 3 times and minced into 1–2 mm pieces. Then the tissue pieces were digested with 2 ml GEXSCOPE^TM^ Tissue Dissociation Solution (Singleron) at 37 °C for 15 min in 15 ml centrifuge tube with sustained agitation. After digestion, using 40-micron sterile strainers to filter the samples and centrifuging the samples at 1000 rpm for 5 m. Then the supernatant was discarded, and the sediment was resuspended in 1 ml PBS (HyClone). To remove the red blood cells, 2 mL GEXSCOPE^TM^ red blood cell lysis buffer (Singleron) was added at 25 °C for 10 m. The solution was then centrifuged at 500×*g* for 5 min and suspended in PBS. The sample was stained with trypan blue (Sigma) and microscopically evaluated.

Single-cell suspensions with 1×10^5^ cells/mL in concentration in PBS (HyClone) were prepared. Single-cell suspensions were then loaded onto microfluidic devices and scRNA-seq libraries were constructed according to Singleron GEXSCOPER protocol by GEXSCOPER Single-Cell RNA Library Kit (Singleron Biotechnologies) [48]. Individual libraries were diluted to 4 nM and pooled for sequencing. Pools were sequenced on Illumina HiSeq X with 150 bp paired end reads.

Raw reads were processed to generate gene expression profiles using an internal pipeline. Briefly, after filtering read one without poly T tails, cell barcode and UMI was extracted. Adapters and poly A tails were trimmed (fastp V1) before aligning read two to GRCh38 with ensemble version 92 gene annotation (fastp 2.5.3a and featureCounts 1.6.2) [49]. Reads with the same cell barcode, UMI and gene were grouped together to calculate the number of UMIs per gene per cell. The UMI count tables of each cellular barcode were used for further analysis.

### Single-cell RNA-Seq data processing

Here, we applied Seurat package to normalize and scale the single-cell gene expression matrix [50, 51]. It was first normalized by “NormalizeData” function with setting normalization method as “LogNormalize”. The uninteresting variations were removed by implementing by “ScaleData” function. Finally, the corrected expression matrix was used as an input for further analysis.

### Cell filtration for mouse scRNA-seq data

Due to the different QC for Immune and breast cells, we adopted two different criteria. The quality of cells was assessed based on three metrics step by step: [1] The number of total UMI counts per cell (library size); [2] The number of detected genes per cell; [3] The proportion of mitochondrial gene counts. After identification of epithelial cells and immune cells in Fig. 1b, Low-quality epithelial cells were filtered if the quality of the cell does not meet the following standards: “total counts: > 5,000; number of genes: >2,000; the proportion of mitochondrial gene counts: <8%”. The criterion for the immune cell is “total counts: >1000; number of genes: >500; the proportion of mitochondrial gene counts: <8%”.

### Cell filtration for human scRNA-seq data

Low-quality cells were filtered if the quality of the cells did not meet the following criteria: “number of genes: >400; number of genes: <7000; the proportion of mitochondrial gene counts: <20%”.

### Dimension reduction, cell clustering, and annotation

We selected the top 3000 largest variable genes as highly variable genes (HVGs) and performed the subsequent analysis such as PCA clustering, WGCNA network analysis and construction of single-cell trajectories based on this set of HVGs. The expression profile of HVGs was centered and scaled values. It was implemented by “FindVariableGenes” function with default parameters. We then used the “RunPCA” function to perform the principal component analysis (PCA). The number of significant principal components was determined by “jackstraw” function. The analysis identified 50 significant principal components to supply to the “RunPCA” function. We then utilized the “FindClusters” function to conduct the cell clustering with resolution setted as 0.4. We annotated cell clusters based on the expression of curated known cell markers on t-SNE plot.

### Differential expression analysis

Differential gene expression analysis was performed by the “FindMarkers” function. The statistical method to identify differentially expressed genes was based on Wilcox rank sum test. The “FindMarkers” function was run with default parameters.

### Survival analysis and enrichment analysis

The survival analysis was performed on KMplot (http://kmplot.com/analysis/index.php?p=service&cancer=breast) [52]. We supplied multiple gene names to the KMplot by using “use mean expression of selected genes” option. We restricted our analysis in Luminal B type patients. The enrichment analysis was performed on MsigDB (https://www.gsea-msigdb.org/gsea/msigdb) with default parameters [53, 54]. Gene expression profile of breast cancer patient was downloaded from the TCGA website (https://portal.gdc.cancer.gov/). Raw reads counts were extracted from files with the suffix “htseq.counts”. The trimmed mean of M-values (TMM) normalized expression value was generated by “edgeR” package [55]. The clinical information was also downloaded from the TCGA website. The information of cancer subtype was retrieved from TCGA previous study [24].

### Cancer signature scoring

The signature genes were the genes differentially expressed between MMTV-PyMT and control FVB mouse at week 17 in bulk RNA-seq. The edgeR package in R was applied to identify differentially expressed genes [55]. Genes with FDR < 0.05 and expression fold change >1.5 (upregulated genes) or <0.67 (down-regulated genes) were defined as differentially expressed genes. Cancer signature scoring was calculated by subtracting the mean expression of the down-regulated genes from the mean expression of the upregulated genes [14, 43]. We defined cancer and normal cells by the median score based on cancer signature genes. Cells with score lower than the median were defined as normal cells and cells with score higher than the median as cancer cells.

### Gene set variation analysis (GSVA)

Pathway analyses were predominantly performed on the gene sets described in the Molecular Signatures Database (MSigDB) and exported using the GSEABase package (version 1.36.0). To reduce pathway overlaps and redundancies, genes associated with multiple gene sets were trimmed from these gene sets and thus retained genes are only associated with one gene set by following previous study [56]. Most gene sets retained >70% of the associated genes. Next, to assign stemness of cancer stem-like cell estimates to individual cells. We applied GSVA using standard settings, as implemented in the GSVA package (version 1.22.4). Differences in pathway activities scored per cell by GSVA between the different clusters.

### Reconstruction of differentiation trajectories using Monocle

Using the R package Monocle (version 2.8.0), differentiation hierarchies within different clusters were reconstructed [57]. Cell fate decisions and differentiation trajectories were reconstructed with the Monocle 2 package, which utilized reverse graph embedding based on a user-defined gene list to generate a pseudo-time plot that could account for both branched and linear differentiation processes.

## Supplementary information

Figure 1S

Figure 2S

Figure 3S

Figure 4S

Figure 5S

Table S1

Table S2

Table S3

Table S4

Table S5

Table S6

Table S7

Supplementary figure legends

## Data Availability

The scRNA-seq data for MMTV-PyMT mouse has been deposited in the NCBI under the accession code PRJNA762594. The bulk RNA-seq data for MMTV-PyMT and wild-type control mouse has been deposited in the NCBI under the accession code PRJNA761912. The scRNA-seq data for breast cancer patients have been deposited in the NCBI under the accession code PRJNA764023.
