# Supplementary material for: Single-cell transcriptomics reveal the heterogeneity and dynamic of cancer stem-like cells during breast tumor progression
Source: Cell Death Dis. 2021 Oct 21;12(11):979. doi: 10.1038/s41419-021-04261-y (PMC8531288; doi:10.1038/s41419-021-04261-y)

a

Sample	Cells
Week 07	5,996
Week 09	9,588
Week 11	4,925
Week 17	11,269

b Cell Filtration

Cell Types	Total Counts	Genes	Percent_mt	Cell Counts
Immune Cells	>1,000	>5,00	< 8%	8,954
Epithelial Cells	>5,000	>2,000	< 8%	12,039

c Breast epithelial cells before filtering / Breast epithelial cells after filtering

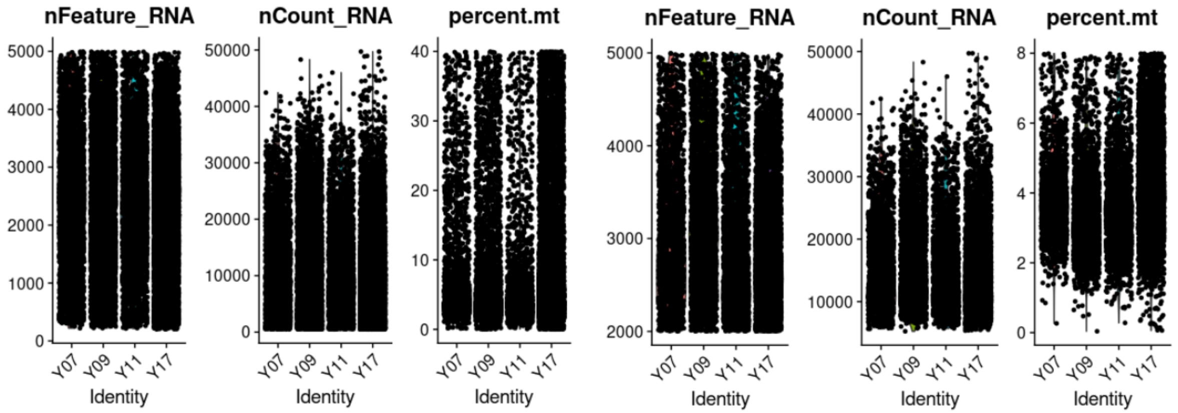

d Immune cells before filtering / Immune cells after filtering

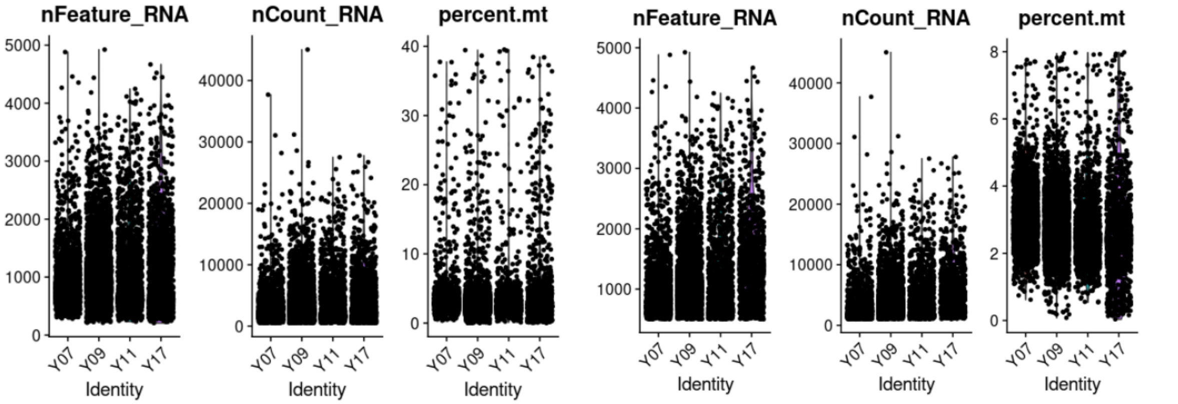

Supplement: Supplementary file 1 — Figure 1S [file 41419_2021_4261_MOESM1_ESM.pdf]
