# Supplementary figures and images for: Single-cell transcriptomics reveal the heterogeneity and dynamic of cancer stem-like cells during breast tumor progression
Source: Cell Death Dis. 2021 Oct 21;12(11):979. doi: 10.1038/s41419-021-04261-y (PMC8531288; doi:10.1038/s41419-021-04261-y)

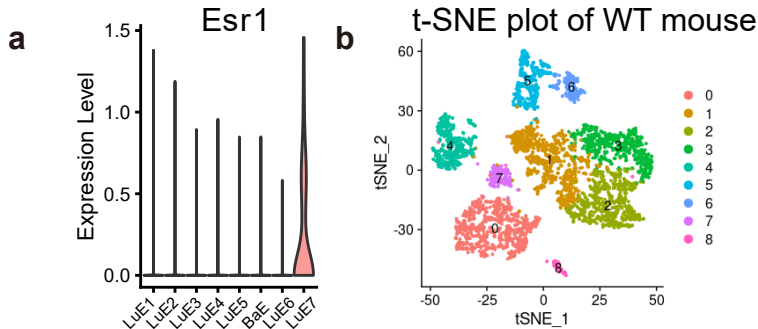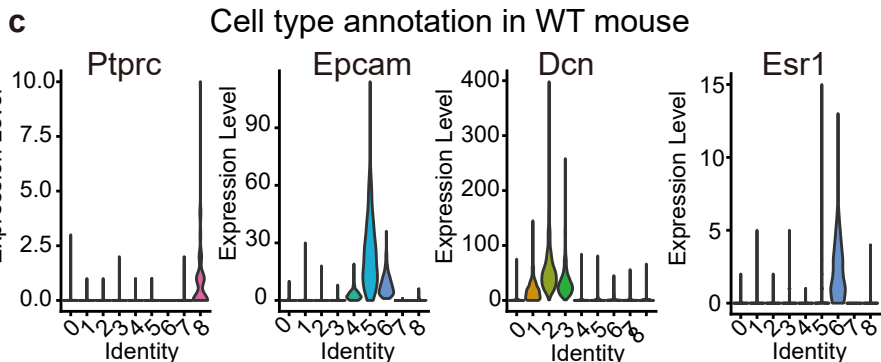

**d**

| Cluster | 0   | 1   | 2   | 3   | 4   | 5   | 6   | 7   | 8  |
|---------|-----|-----|-----|-----|-----|-----|-----|-----|----|
| Counts  | 878 | 843 | 705 | 594 | 467 | 386 | 184 | 165 | 76 |

Supplement: Supplementary file 2 — Figure 2S [file 41419_2021_4261_MOESM2_ESM.pdf]

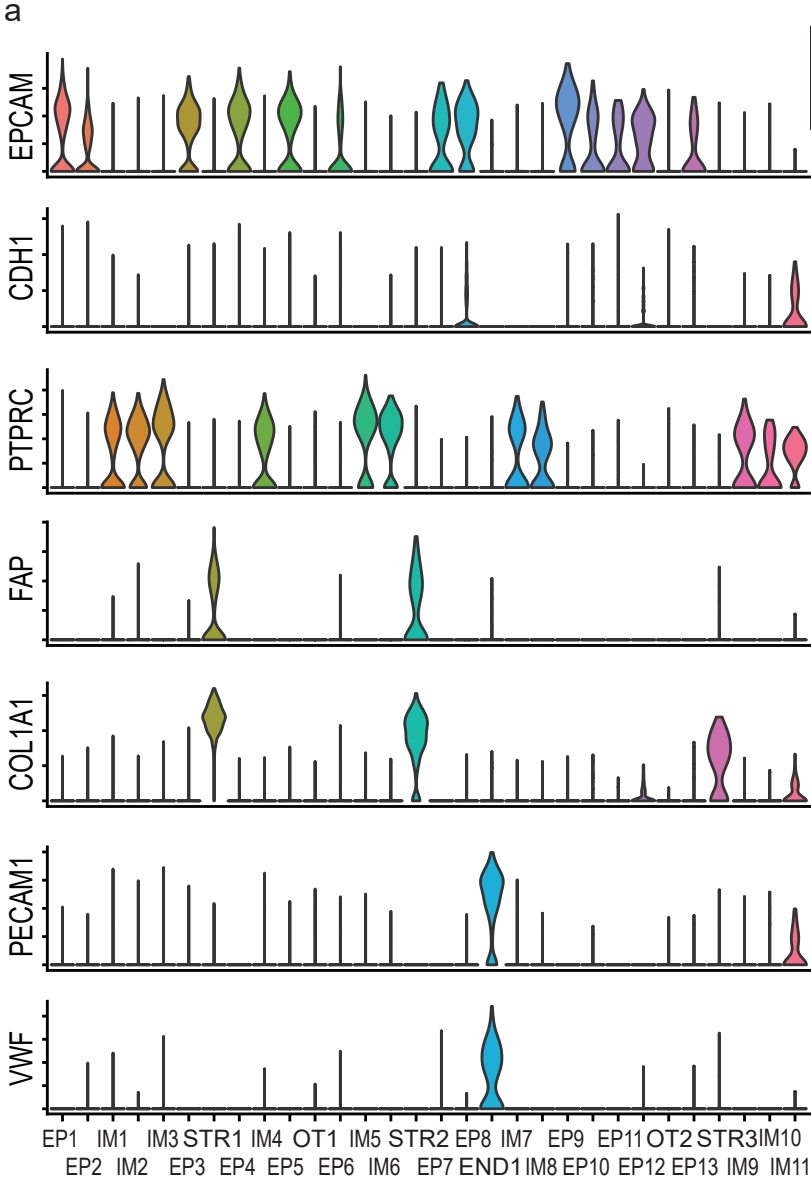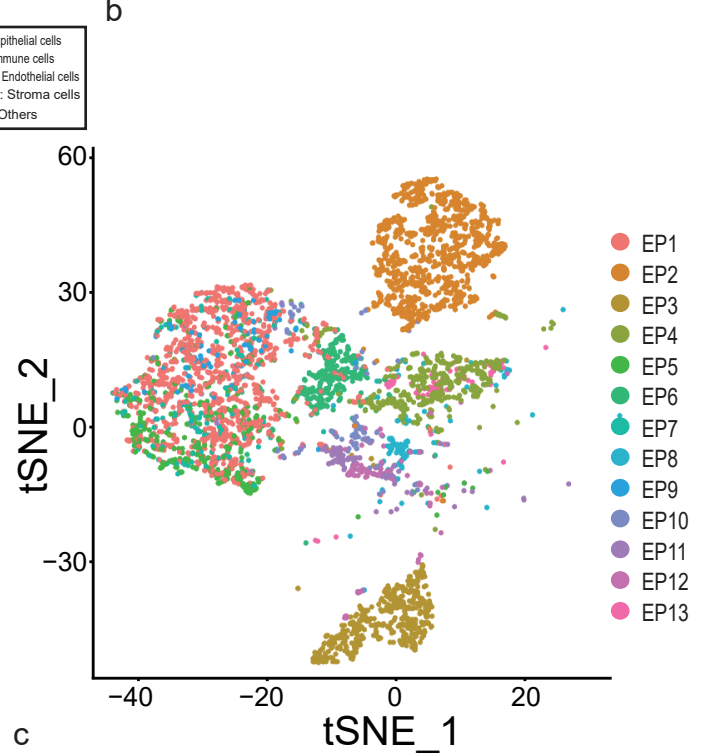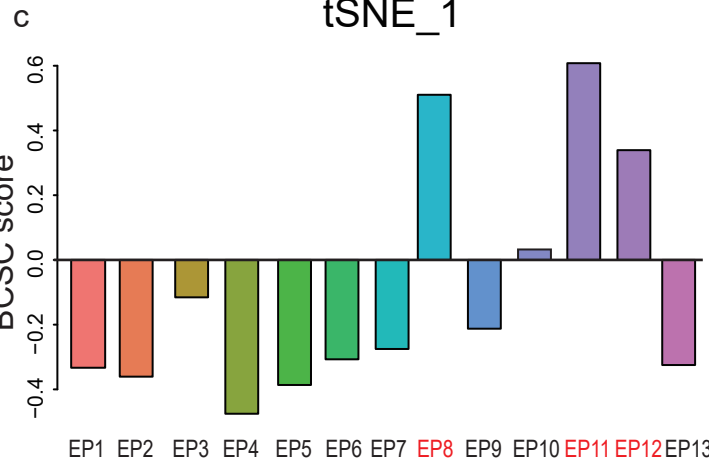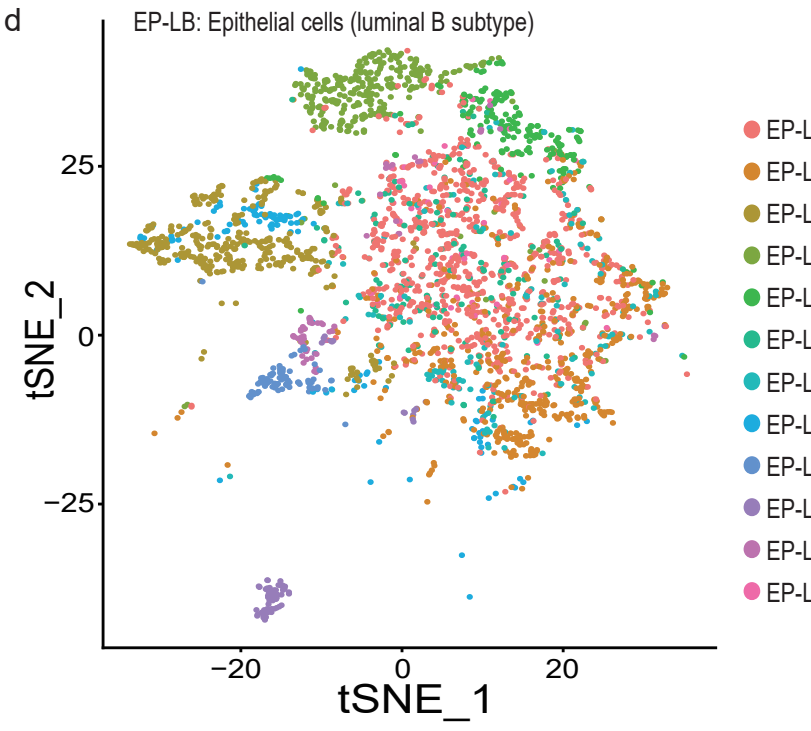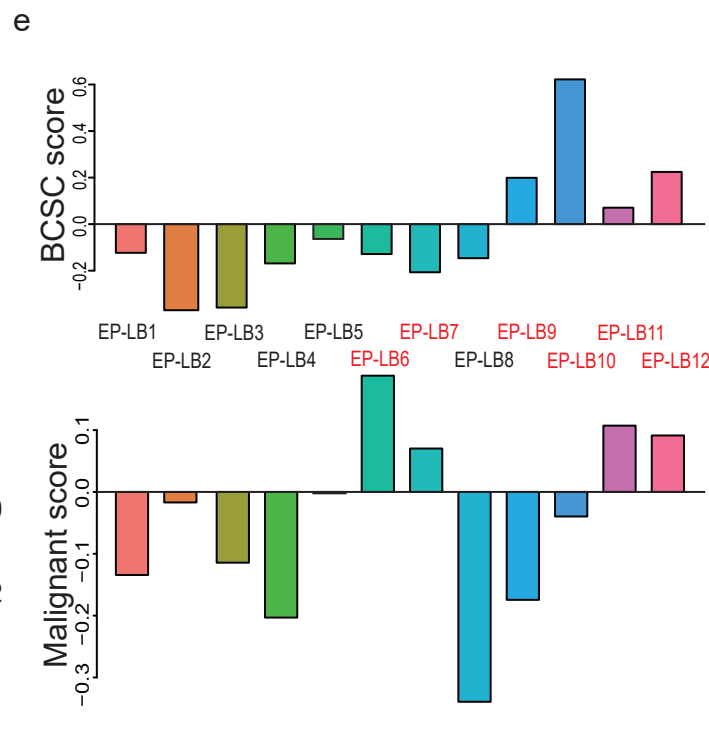

Supplement: Supplementary file 4 — Figure 4S [file 41419_2021_4261_MOESM4_ESM.pdf]

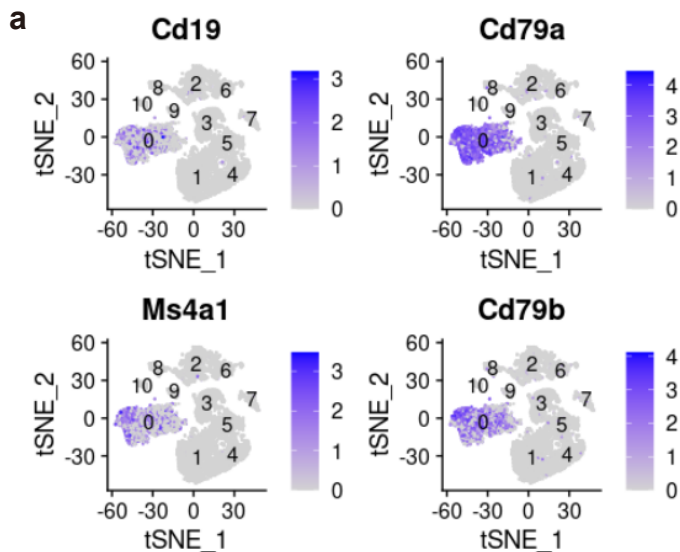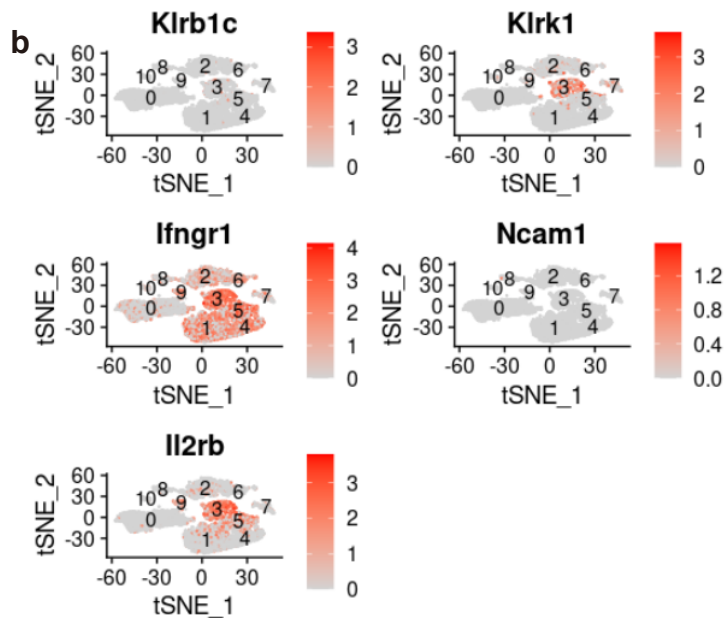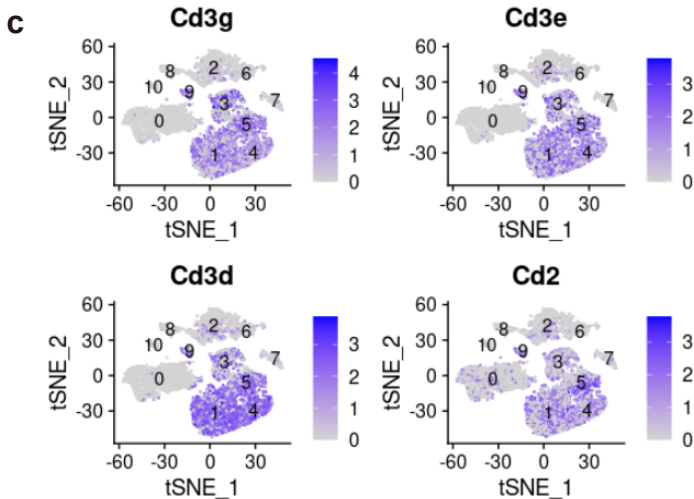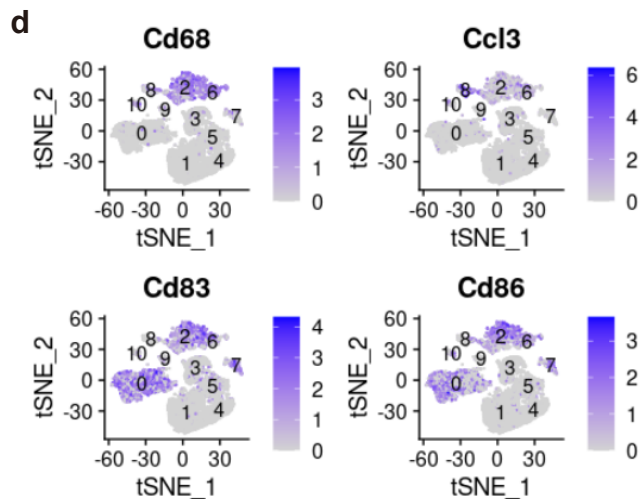

Supplement: Supplementary file 5 — Figure 5S [file 41419_2021_4261_MOESM5_ESM.pdf]
