# Supplementary material for: Single-cell transcriptomics reveal the heterogeneity and dynamic of cancer stem-like cells during breast tumor progression
Source: Cell Death Dis. 2021 Oct 21;12(11):979. doi: 10.1038/s41419-021-04261-y (PMC8531288; doi:10.1038/s41419-021-04261-y)

a

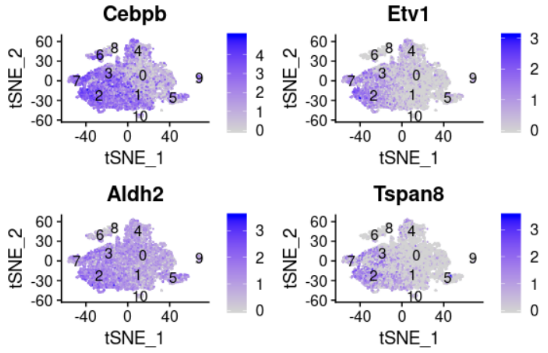

b Stemness associated gene list used by GSVA algorithm

Marker Used	References
Aldh3a2	PMID: 30220009
Aldh1a3	PMID: 31340763
Aldh2	PMID: 31480503
Cd24a	PMID: 31340763
Itgb1	PMID: 27197172
Itgb6	PMID: 31340763
Epcam	PMID: 31340763
Cd14	PMID: 21930782
Kit	PMID: 28719381
Prom1	PMID: 18241344
Ly6a	PMID: 19048122
Cd44a	PMID: 26516409
Mme	PMID: 28719381
Itgb3	PMID: 31555586
Sox9	PMID: 30206184
Nectin4	PMID: 25940879
Flot2	PMID: 19951611
Vegta	PMID: 21802218

c

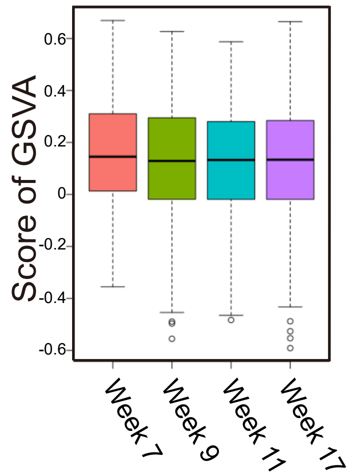

d

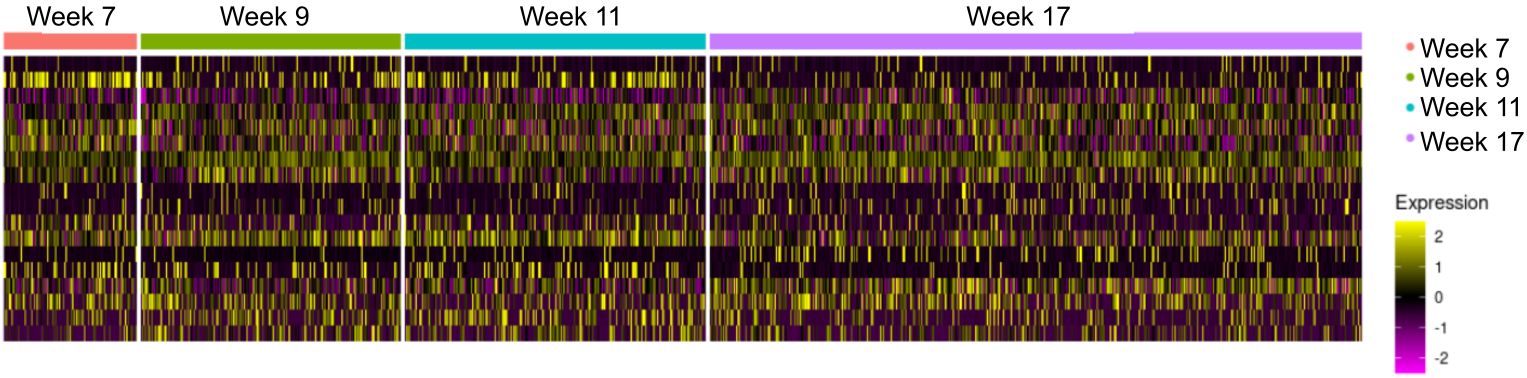

Supplement: Supplementary file 3 — Figure 3S [file 41419_2021_4261_MOESM3_ESM.pdf]
