# Supplementary material for: Single-cell transcriptomics reveal the heterogeneity and dynamic of cancer stem-like cells during breast tumor progression
Source: Cell Death Dis. 2021 Oct 21;12(11):979. doi: 10.1038/s41419-021-04261-y (PMC8531288; doi:10.1038/s41419-021-04261-y)
Supplement: Supplementary file 13 — Supplementary figure legends [file 41419_2021_4261_MOESM13_ESM.docx]

### Fig. S1: Data quality control and cell type annotation

**a** A table summarizing the cell counts for each tumor progression stage. **b** Different filer [criterion](javascript:;) of immune cells and epithelial cells were shown in the table. **c, d** Violin plot showing the distribution of nFeature_RNA, nFeature_count and percentage of reads mapping to the MT genes for each epithelial cell (c) and immune cell (d) before and after quality control.

### Fig. S2: Annotation of ER^high^ and ER^low^ Luminal Cells

**a** Violin plots showing the expression level of indicated genes among cell clusters. **b** t-SNE plot of the cells in wild-type (WT) mice colored by the clusters at age of week 12. **c** Violin plots showing the expression level of the marker genes, Ptprc (immune cells), Epcam (epithelial cells), Dcn (endothelial cells) and Esr1 (ER^high^ Luminal Cells). **d** A table summarizing the cell number of the corresponding clusters.

### Fig. S3: The stemness associated gene list and cancer stemness score

**a** t-SNE plot of the epithelial cells was colored by the expression level of indicated genes, Cebpb, Etv1, Aldh2 and Tspan8. **b** Stem cell markers used to calculate stemness score by GSVA algorithm. **c** GSVA score for each tumor progression stage in cluster LuE2 revealed by bar plot. **d** Heatmap of expression profile of stem cell marker genes in each tumor progression stage in cluster LuE2.

### Fig. S4: The identification and annotation of BCSCs and malignant cells among epithelial cells from breast cancer patients

**a** Violin plot showed the expression of marker genes in each cluster. **b** t-SNE plot demonstrated the distribution of epithelial cells of all six breast cancer patients. **c** Histogram showed the BCSC score for each epithelium cluster. The clusters with high BCSC score were defined as BCSC clusters and the name of these BCSC clusters were highlighted in red. **d** t-SNE plot showed the distribution of epithelial cells from three luminal B breast cancer patients. **e** Histogram showed the BCSC and malignant score for each epithelium cluster from three luminal B breast cancer patients. The clusters with high BCSC or malignant score were defined as BCSC or malignant clusters respectively and the name of these clusters were highlighted in red.

### Fig. S5: The identification and annotation of infiltrated immune cell types in MMTV-PyMT gland pad

**a-d** The t-SNE plot of the immune cells colored by the expression level of indicated marker genes in B cells (a), Natural killer cells (b), T cells (c) and Macrophages (d)
